# O Outro Lado da Moeda: Os Riscos da Discussão de Dados Médico-Científicos pela Mídia Durante a Pandemia de COVID-19

**DOI:** 10.36660/abc.20200449

**Published:** 2020-08-19

**Authors:** Caio Julio Fernandes, Fernando Ganem, Fabio Gravina Olivieri, Marcelo Fadul Vilibor, Alfredo Salim Helito

**Affiliations:** 1 Universidade de São Paulo Departamento de Cardiopneumologia São Paulo SP Brasil Universidade de São Paulo-Departamento de Cardiopneumologia,São Paulo, SP - Brasil; 2 Hospital Sírio-Libanês São Paulo SP Brasil Hospital Sírio-Libanês, São Paulo, SP - Brasil

**Keywords:** Coronavirus, COVID-19, Pandemia, Quarentena Isolamento Social, Doenças Respiratórias, Doenças Transmissíveis, Diagnóstico Diferencial, Tecnologia da Informação/tendências, Mídias Sociais

A pandemia de COVID-19, assim determinada pela ONU em março de 2020, trouxe uma série de mudanças no cotidiano de toda a população. Medidas de isolamento social, quarentena e *lockdown* foram implementadas em diversos países. O número elevado de casos, já próximo de 4 milhões no mundo,^1^ com mais de 250.000 mortos pela doença, fez com que houvesse um grande interesse na patologia, e uma verdadeira revolução na produção e divulgação de dados médicos ocorreu por conta disso. Uma grande quantidade de artigos científicos avaliando todos os aspectos de COVID-19, desde a sua epidemiologia, passando pelo seu quadro clínico e por potenciais possibilidades terapêuticas tornou-se disponível para a comunidade médica.^2^ Em pouco mais de 4 meses mais de 10.000 artigos foram publicados sobre o tema e, de forma inédita, disponibilizados gratuitamente, em tempo real, pelos principais periódicos da literatura médica.

Essa velocidade de produção e imensa quantidade de dados disponíveis não vêm sem um preço. Boa parte destes artigos não passou por uma revisão de metodologia adequada, sequer foi avaliada por pares e não foi depurada pelo tempo. A necessidade de compreender a COVID-19 e de buscar melhores alternativas terapêuticas fez com que houvesse uma avalanche de estudos questionáveis. O joio misturou-se ao trigo e recomendações médicas passaram a mudar com uma velocidade assustadora. Dados com maiores graus de confiabilidade e de evidência, advindos de estudos aleatorizados e controlados por placebo passaram a ser considerados demorados demais. Séries de casos e opiniões de especialistas começaram a pautar condutas clínicas, com impacto direto no manejo clínico dos pacientes. Ao invés de demonstrarem soluções, a inundação de estudos passou a ser um problema e a gerar confusão para a prática clínica no manejo dos pacientes com COVID-19.

Tomemos a avaliação da anticoagulação dos pacientes com COVID-19 como exemplo. Dados bastante consistentes da literatura sugerem que haja uma patologia vascular nos pulmões dos pacientes vítimas de quadros respiratórios graves de COVID-19. Uma alta incidência de trombose foi identificada nesta população, maior do que em outras situações de gravidade clínica semelhante, mesmo na vigência de anticoagulação profilática adequada,^3^ Trombos foram identificados na circulação pulmonar, em vasos de pequeno calibre, não identificáveis na angio-tomografia convencional.^4^ A elevação do D-dímero demonstrou impacto na mortalidade de pacientes com COVID-19, sugerindo que pacientes com quadros trombóticos mais graves na microcirculação apresentavam pior prognóstico.^5^ Finalmente, a avaliação da mecânica pulmonar dos pacientes com insuficiência respiratória por COVID-19 demonstrou que a complacência pulmonar desta população não era tão reduzida quanto o esperado. No entanto, havia na mesma população uma elevação surpreendente da fração de “shunt” pulmonar, denotando que boa parte da hipoxemia não se devia a alterações da ventilação (como esperado em outras formas de síndrome do desconforto respiratório agudo), e sim às alterações da circulação pulmonar.^6^

Assim, se há uma patologia trombótica da circulação pulmonar em uma doença grave, faz sentido intuitivo o uso de anticoagulantes para o tratamento dessa condição e a melhora potencial da hipoxemia e da troca gasosa. Séries de casos e estudos retrospectivos demonstraram que haveria um benefício clínico potencial palpável com essa conduta.^7^ No entanto, doses adequadas, melhores agentes a serem empregados e intensidade de coagulação não podem ser definidos por essas modalidades de estudos. Apenas estudos prospectivos aleatorizados e controlados podem prover a evidência necessária para que os pacientes possam ser tratados com segurança, definindo de forma precisa essas questões. Entretanto, enquanto esses estudos não são terminados e estes dados não se tornam disponíveis, vários consensos fazem recomendações muito distintas e, por vezes, contraditórias sobre qual a melhor forma de promover a anticoagulação nos pacientes com COVID-19 (seja ela profilática, terapêutica ou ainda com “esquemas alternativos”)^8 - 10^ Múltiplas orientações acabam por gerar confusão e insegurança para os médicos, e cautela é fundamental para a interpretação destas informações.

Todavia, há um terceiro componente que, nestes tempos de COVID-19, interpõe-se entre a informação médica, sua interpretação por um médico, e sua transmissão para o paciente: a mídia jornalística. O grande interesse da população por informações sobre a COVID-19 fez com que houvesse uma intensa cobertura pela imprensa de todos os aspectos da doença, inclusive de avanços terapêuticos. Porém, via de regra, a informação passada diretamente do artigo científico pelo jornalista para a população carece de interpretação, de crítica e da avaliação de riscos. E o benefício de levar a informação pode ser suplantado pelo risco que essa informação sem crítica possa causar, caso acarrete uma conduta clínica.

**Figura 1:**
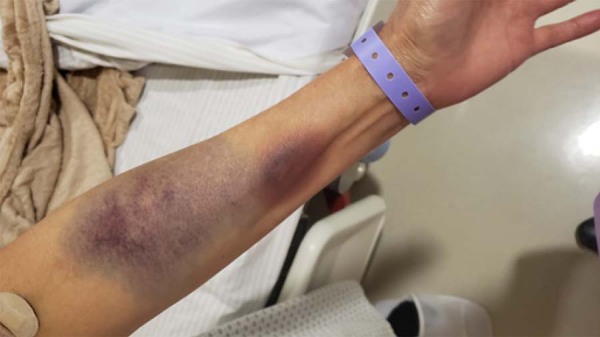
– Hematomas espontâneos em uma paciente de 62 anos, que fez uso de ácido acetil salicílico, rivaroxabana e varfarina para se proteger da gravidade de uma eventual infecção por COVID-19 (entretanto, seu PCR e sorologia vieram negativos). À entrada seu INR era 26 e o R do TTPA de 2. Com a reversão da anticoagulação e observação clínica, a paciente não apresentou outras complicações hemorrágicas.

Considere esse exemplo: uma paciente de 62 anos dá entrada na emergência de um hospital com quadro de grandes hematomas ao longo do corpo, de origem espontânea ( Figura 1 ). Quinze dias antes ela iniciara quadro de rinorréia hialina sem febre ou mialgia. Temendo a COVID-19 ela buscara informações sobre a patologia e encontrara dados na mídia sobre um potencial tratamento com anticoagulantes. Ela então buscou se proteger da COVID-19 utilizando diversos anticoagulantes concomitantes. Começara o uso, por conta própria, de rivaroxabana, varfarina e ácido acetil-salicílico. Por via das dúvidas, utilizara também hidroxicloroquina e azitromicina (também influenciada por dados da mídia jornalística, que destacaram estudos com potencial benefício destas terapêuticas).^11^ À entrada, apresentava hemoglobina de 12, INR de 26 e TTPA com R de 2. Foi internada e a anticoagulação foi revertida. Submetida tanto ao PCR para busca do SARS-CoV-2 (causador da COVID-19) quanto à pesquisa sorológica, ambas resultaram negativas. Assim, esta paciente nunca teve COVID-19, mas poderia ter morrido por complicações de terapias ainda em avaliação para o tratamento de uma doença que ela nunca teve. A COVID-19 foi identificada há apenas 5 meses. Por mais grave que ela seja, por maior que seja o número de vítimas, há a necessidade de tempo e experiência, tanto para seu manejo clínico^12^ quanto para a interpretação de dados científicos produzidos em quantidades e velocidades nunca antes vistas. A democratização da informação é fundamental, e esse papel é feito com excelência pela imprensa. No entanto, informações técnicas brutas, sem a necessária depuração conferida pela experiência clínica podem ter consequências bastante deletérias, ao serem absorvidas sem cuidado por uma população fragilizada pelo receio da doença. O acesso à informação, providenciado pela mídia é fundamental para que o paciente possa participar ativamente do seu tratamento. Entretanto, este tratamento sempre deve ser orientado pelo profissional mais capacitado para fazê-lo, o médico.
