# Supplementary material for: O Outro Lado da Moeda: Os Riscos da Discussão de Dados Médico-Científicos pela Mídia Durante a Pandemia de COVID-19
Source: Arq Bras Cardiol. 2020 Aug 19;115(2):278–80. [Article in Portuguese] doi: 10.36660/abc.20200449 (PMC8384294; doi:10.36660/abc.20200449)
Supplement: Translation

# Translation: The Other Side of the Coin: Risks of Media Discussions of Scientific Medical Data During the COVID-19 Pandemic

**Authors:** Caio Julio Fernandes, Fernando Ganem, Fabio Gravina Olivieri, Marcelo Fadul Vilibor, Alfredo Salim Helito

**Affiliations:** 1 Brazil Universidade de São Paulo-Departamento de Cardiopneumologia, São Paulo, SP - Brazil; 2 Brazil Hospital Sírio-Libanês, São Paulo, SP - Brazil

**Keywords:** Coronavirus, COVID-19, Pandemics, Quarantine, Social Isolation, Respiratory Diseases, Communicable Diseases, Diagnostic, Differencial, Information Technology/trends, Social Media

The COVID-19 declared by the WHO in March 2020 has brought about a series of changes in the population’s daily life. Social isolation measures, quarantine, and lockdown have been implemented in diverse countries around the world. The elevated number of cases, currently close to 4 million worldwide,^1^ with more than 250,000 deaths due to the disease, has led to great interest in pathology; on account of this, a revolution has occurred in the production and publication of medical data. Numerous scientific articles evaluating all aspects of COVID-19, from epidemiology to clinical presentation and potential therapeutic options, have become available to the medical community.^2^ In a little over 4 months, more than 10,000 articles have been published on this topic, and, in a never-before-seen manner, the leading medical journals have made them available in real time, free of charge.

This speed of production and this immense quantity of available data do not come without a price. Many of these articles were not submitted to adequate review of methodology; much less were they evaluated by their peers or refined over time. The need to understand COVID-19 and to search for better alternative therapies has led to an avalanche of questionable studies. The chaff has been mixed with the wheat, and medical recommendations have started to change at frightening speeds. Data with higher degrees of reliability and evidence, derived from randomized, placebo-controlled studies, are now considered too time-consuming. Case series and expert opinions have begun to guide clinical conduct, with a direct impact on clinical management of patients. Instead of indicating solutions, the flood of studies has become a problem and begun to cause confusion regarding clinical practice for managing patients with COVID-19.

Let us take the evaluation of anticoagulation in patients with COVID-19 as an example. Rather consistent data from the literature have suggested that there is a vascular pathology in the lungs of patients with severe respiratory conditions in COVID-19. A high incidence of thrombosis has been identified in this population, greater than in other similarly severe clinical situations, even under adequate prophylactic anticoagulation.^3^ Thrombi have been identified in pulmonary circulation, in small vessels that are not identifiable on conventional angiography.^4^ Increased D-dimer has shown an impact on mortality of patients with COVID-19, suggesting that patients with more severe thrombotic conditions in their microcirculation have worse prognosis.^5^ Finally, evaluation of pulmonary mechanics of patients with respiratory failure due to COVID-19 has demonstrated that pulmonary compliance was not as reduced as expected in this population. There was, however, a surprising increase in pulmonary shunt fraction in this population, indicating that much of the hypoxemia was not due to changes in ventilation (as expected in other forms of acute respiratory distress syndrome), but rather to changes in pulmonary circulation.^6^

Accordingly, if there is a thrombotic pathology of the pulmonary circulation in a severe disease, it would intuitively make sense to use anticoagulants to treat this condition and potentially improve hypoxemia and the gas exchange. Case series and retrospective studies have demonstrated that there would be a potentially tangible clinical benefit to this conduct.^7^ However, adequate dosages, the best agents to use, and the coagulation intensity cannot be defined by these types of studies. Only prospective randomized controlled studies can provide the evidence that is necessary in order to treat patients safely, by accurately defining these questions. In the meantime, while these studies have not been completed and data are not yet available, several consensuses have made very different and, at times, contradictory recommendations regarding the best way to promote anticoagulation in patients with COVID-19 (be it prophylactic, therapeutic, or via “alterative regimens”).^8 - 10^ Multiple orientations often end up causing confusion and insecurity on the part of physicians, and caution is fundamental when interpreting this information.

There is, moreover, a third component which, during the COVID-19 pandemic, stands between medical information, physicians’ interpretation thereof, and communication with a patient, namely, journalistic media. The population’s growing interest in information about COVID-19 has led to intense coverage on the part of the press, regarding all aspects of the disease, including therapeutic advances. However, information communicated directly from a scientific article to the population by journalists, as a rule, requires interpretation, criticism, and risk assessment. The benefit of taking this information in may be outweighed by the risk that this information, without criticism, may induce, in the event that it results in clinical conduct.

Consider the following example: A 62-year-old female patient was admitted to the emergency room with large spontaneous hematomas throughout her body ( Figure 1 ). Fifteen days prior, she had onset of hyaline rhinorrhea, without fever or myalgia. Fearing COVID-19, the patient sought information about pathology, and she came across some data in the media about a potential treatment with anticoagulants. She subsequently attempted to protect herself from COVID-19 using diverse anticoagulants concomitantly. Of her own accord, she began taking rivaroxaban, warfarin, and acetylsalicylic acid. Just in case, she had also taken hydroxychloroquine and azithromycin (also influenced by data from journalistic media, which mentioned studies on the potential benefits of these therapies.^11^ ) Upon admission, she presented hemoglobin 12, international normalized ratio 26, and activated partial thromboplastin time ratio 2. She was hospitalized, and anticoagulation was reversed. She underwent both PCR and serology for SARS-CoV-2 (which causes COVID-19), both of which were negative. In this manner, this patient, who had never had COVID-19, might have died due to complications from therapies which are still being evaluated for treating a disease which she never had. COVID-19 was identified only 5 months ago. Notwithstanding its severity and the high number of victims, time and experience are still necessary, in respect to both clinical management^12^ and interpretation of scientific data produced in unprecedented quantities and speeds. The democratization of information is fundamental, and the press is doing an excellent job in this role. Raw technical information, however, without the necessary refinement provided by clinical experience, may have very harmful consequences if it is carelessly absorbed by a population that is fragile due to concerns about this disease. Access to information provided by the media is fundamental in order for patients to participate actively in their treatment. These treatments, however, should always be guided by the professionals who are most qualified to conduct them, namely physicians.

**Figure 1:** – *Spontaneous hematomas in a 62-year-old patient who used acetylsalicylic acid, rivaroxaban and warfarin to protect herself from COVID-19 (however, her PCR and serology tests were both negative). At the Emergence Room, her INR was 26 and the activated partial thromboplastin time ratio was 2. With the reversal of anticoagulation and clinical observation, the patient had no other bleeding complications.*
